# Fast Visual Odometry for a Low-Cost Underwater Embedded Stereo System †

**DOI:** 10.3390/s18072313

**Published:** 2018-07-17

**Authors:** Mohamad Motasem Nawaf, Djamal Merad, Jean-Philip Royer, Jean-Marc Boï, Mauro Saccone, Mohamed Ben Ellefi, Pierre Drap

**Affiliations:** Aix-Marseille Université, CNRS, ENSAM , Université De Toulon, LIS UMR 7020, Domaine Universitaire de Saint-Jérôme, Bâtiment Polytech, Avenue Escadrille Normandie-Niemen, 13397 Marseille, France; Djamal.Merad@univ-amu.fr (D.M.); Jean-Philip.Royer@univ-amu.fr (J.-P.R.); Jean-Marc.Boi@univ-amu.fr (J.-M.B.); Mauro.Saccone@univ-amu.fr (M.S.); Mohamed.Ben-Ellefi@univ-amu.fr (M.B.E.)

**Keywords:** image processing, underwater imaging, embedded systems, stereo vision, visual odometry, 3D reconstruction

## Abstract

This paper provides details of hardware and software conception and realization of a stereo embedded system for underwater imaging. The system provides several functions that facilitate underwater surveys and run smoothly in real-time. A first post-image acquisition module provides direct visual feedback on the quality of the taken images which helps appropriate actions to be taken regarding movement speed and lighting conditions. Our main contribution is a light visual odometry method adapted to the underwater context. The proposed method uses the captured stereo image stream to provide real-time navigation and a site coverage map which is necessary to conduct a complete underwater survey. The visual odometry uses a stochastic pose representation and semi-global optimization approach to handle large sites and provides long-term autonomy, whereas a novel stereo matching approach adapted to underwater imaging and system attached lighting allows fast processing and suitability to low computational resource systems. The system is tested in a real context and shows its robustness and promising future potential.

## 1. Introduction

Mobile systems nowadays are undergoing a growing need for self-localization to determine their absolute/relative position over time accurately. Despite the existence of very efficient technologies that can be used on-ground (indoor/outdoor), such as the Global Positioning System (GPS), optical signals, and radio beacons, in the underwater context, most of these signals are jammed so that similar techniques cannot be used. On the other hand, solutions based on active acoustics, such as imaging sonars, water linked GPS or Doppler Velocity Log (DVL) devices remain expensive and require high technical skills for deployment and operation. Moreover, their size specifications prevent their integration within small mobile systems or even the ability to be handheld. The research for an alternative is ongoing; notably, recent advances in embedded systems have led to relatively small, powerful and cheap devices. This opens promising potential to adopt a light visual odometry approach that provides a relative trajectory in real-time using image sensors, and this describes our main research direction. The developed solution is integrated within an underwater archaeological site survey where it plays an important role in facilitating image acquisition.

In underwater survey tasks, mobile underwater vehicles (or divers) navigate over the target site to capture images. The obtained images are treated in a later phase to obtain various information and also to form a realistic 3D model using photogrammetry techniques [1]. In such a situation, the main problem is covering the underwater site totally before ending the mission. Otherwise, we may obtain incomplete 3D models, and the mission cost will rise significantly as further exploitation will bw needed. However, the absence of an overall view of the site, especially under bad lighting conditions, makes the scanning operation blind. In practice, this leads to over-scanning the site which is a waste of time and cost. From another perspective, the quality of the taken images may go below an acceptable limit. This mainly happens in terms of lightness and sharpness, which is often hard to quantify visually on the fly. In this work, we propose solutions for the aforementioned problems. Most importantly, we propose to guide a survey based on a visual odometry approach that runs on a distributed system in real-time. The output ego-motion helps to guide the site scanning task by showing approximate scanned areas. Moreover, overall subjective lightness and sharpness indicators are computed for each image to help the operator control the image quality.

**Figure 1:**
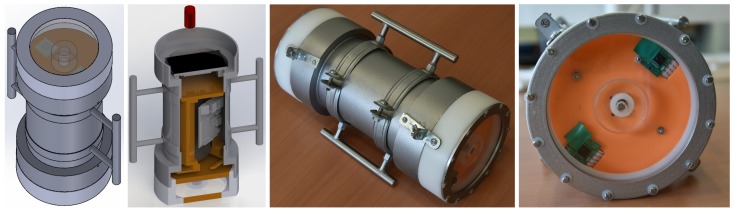
The handheld stereo system design and prototype.

**Figure 2:**
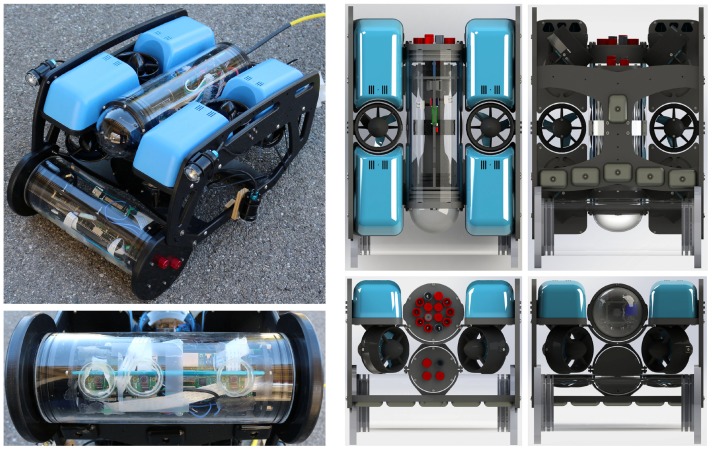
The built trifocal system integrated within a blueROV 2 (the front enclosure).

Overall, we provide a complete hardware and software solution for the problem through the conception and realization of a stereo embedded system dedicated to underwater imaging. Two configurations are considered: first, a handheld system to be used by a diver (see Figure 1), and second, a system attached to a customizable Remotely Operated underwater Vehicle (ROV) from BlueRobotics [2] (see Figure 2). Both configurations share similar main architecture (all provided details are for both configurations unless otherwise stated). The system, equipped with two high definition cameras (three cameras in the ROV-attached configuration), can take and store hardware synchronized stereo images while having long-term autonomy. In contrast to other commercially available off-the-shelf products where the system’s role ends with image storage, the designed system is based on distributed embedded systems with ARM processors and a Linux operating system and is capable of running most image processing techniques smoothly in real-time. The available optimized open source libraries, such as OpenCV [3] and OpenCL [4], allow straightforward extension of the provided functions and full customization of the system to suit different contexts.

In common approaches of visual odometry, a significant part of the overall runtime is spent on feature point detection, description, and matching, whereas another significant part is dedicated to the optimization process, namely, the bundle adjustment (BA) [5] procedure. In the tested baseline algorithm, feature point matching represents ≈65% of runtime in the local/relative bundle adjustment (BA) approach. Despite their accuracy and successful broad application, modern feature descriptors, such as Scale Invariant Feature Transform (SIFT) [6] and Speeded Up Robust Features (SURF) [7], rely on differences of Gaussians (DoG) and fast Hessian, respectively, for feature detection. These methods are two times slower than the traditional Harris detector [8]. Further, the sophisticated descriptors that are invariant to scale and rotation, which is not necessary for stereo matching, slow down the computation. Moreover, brute force matching is often used which is also time-consuming. In our proposed method, we rely on low-level Harris-based detection and a template matching procedure which significantly speeds up the point matching. Further, whereas in traditional stereo matching the search for correspondence is done along the epipolar line within a specific fixed range, in our method, we proceed first by computing, a priori, a rough depth belief based on image lightness and following the law of light divergence over distance. This is only valid for a configuration in which the only light source is fixed to the system, which is the case here. Hence, our first contribution is that we benefit from rough depth estimation to limit the point correspondence search zone to reduce the processing time. It is worth mentioning that even for the surveys in shallow water where the sunlight provides good visibility, it is preferable to wait for sunset before starting the survey because of the sunlight ripple effect on the scanned site [9] which misleads the photogrammetry process, as it disturbs the photometric consistency.

From another perspective, traditional visual odometry methods based on local BA suffer from rotation and translation drift that grow with time [10]. In contrast, solutions based on using features from the entire image set, such as global BA [5], require more computational resources which are very limited in our case. Similarly, simultaneous localization and mapping (SLAM) approaches [11], which are known to detect loop closure, although being efficient in most robotics applications, suffer from a growing processing time [12], or are not suitable for raster scan trajectories such as hierarchical approaches [13,14]. In our method, we adopt a semi-global approach which proceeds in the same way as local methods for optimizing a subset of image frames. However, it differs in terms of selecting the frame subset, as local methods use the Euclidean distance and deterministic pose representation to select frames, but ours represents the poses in a probabilistic manner and uses a divergence measure to select such subset of frames. The uncertainty of each newly-estimated pose is computed using a novel approach that uses a machine learning technique on the simulated pose estimation system. This is handled by a neural network that is trained to handle a wide range of ego-motion vectors. This will be addressed in detail in Section 5.4.

The rest of the paper is organized as follows: We survey related works in Section 2. In Section 3, we describe the designed hardware platform and the two configurations that we used to implement our solution. The image acquisition and the quality estimation procedure are explained in Section 4. Our proposed visual odometry method is presented in Section 5. The analytical results of the underwater experiments are presented in Section 6. Finally, we present a summary and conclusions. We note that parts of this work have been presented in [15,16].

## 2. Related Works

In this section, we review related works concerning the two aspects that we mainly aim to improve in our framework: feature point matching and ego-motion estimation.

### 2.1. Feature Point Matching

Common ego-motion estimation methods rely on feature point matching between several poses [17,18,19,20,21,22,23,24]. Real-time methods tend to use fast feature detectors. The most popular are Features from Accelerated Segment Test (FAST) [25], as in [19,20,23], and Harris-based [26], as in [18,21,22]. These types of detectors are frequently associated with patch descriptors. In general, the choice of approach for matching feature points depends on the context. For instance, feature matching between freely-taken images (six degrees of freedom) with baseline toleration has to be invariant to scale and rotation changes. Scale Invariant Feature Transform (SIFT) [6] and the Speeded Up Robust Features (SURF) [7] are well used in this context [17,24,27,28]. In this case, besides being more computationally expensive, the search for a point’s correspondence is generally done using brute force matching.

A new family of feature descriptors that aims to accelerate the extraction process makes use of binary representation computed from image intensity differences tests. The Binary Robust Independent Elementary Features (BRIEF) method [29] is the first in this direction. The method measures the intensity difference on a fixed chosen location pairs around the keypoints which are commonly detected using FAST. An improvement to BRIEF is the Binary Robust Invariant Scalable Keypoints (BRISK) [30], which adds scale and rotation invariance features. This is achieved by introducing multi-scaling and using regular circular pattern around the keypoint. Another difference to BRIEF is that BRISK proposes its own detector, an extension of the AGAST detector [31] (based on FAST) that performs a scale-space search for saliency. Overall, using this over-sampled representation of the keypoint neighborhood makes these methods more sensitive to noise. As this does not has significant inference on terrestrial images, underwater images suffer mostly from turbidity and dust which makes the use of these methods less robust based on our experiments.

In certain situations, some constraints can be imposed to facilitate the matching procedure, in particular, limiting the correspondence search zone. For instance, in the case of pure forward motion, where the focus of expansion (FOE) is a single point in the image, the search for the correspondence of a given point is limited to the epipolar line [32]. Similarly, in the case of sparse stereo matching, the correspondence point lies on the same horizontal line in the case of a rectified stereo or on the epipolar line otherwise. This speeds up the matching procedure, firstly by having fewer comparisons to perform and secondly because low-level features can be used [33,34]. According to our knowledge, no method proposes an adaptive search range following a rough depth estimation from lightness in underwater imaging. We refer to [8] for a comprehensive study of feature point detection and matching.

It is worth mentioning that direct visual odometry methods are well-established when a depth map is available, such as using RGB-D cameras [35]. These featureless methods use geometry transformation between rigid objects in several views to infer ego-motion. Methods that deal with stereo cameras proceed by computing a dense depth estimation that is used to establish a relationship between objects within adjacent views [36], whereas monocular methods [37,38] use a variational approach for estimating pixel-wise depth. The problem is solved under convex assumption using GPU. The main inconvenience of those approaches is the required high computational power and the small baseline between adjacent images which are hard to guarantee in our context.

### 2.2. Ego-Motion Estimation

Estimating the ego-motion of a mobile system is an old problem in computer vision. Two main categories of methods are developed in parallel, namely, simultaneous localization and mapping (SLAM) [34] and visual odometry [18]. In the following text, we highlight the main characteristics of both approaches.

The SLAM family of methods uses probabilistic models to handle a vehicle’s pose. Although this kind of method was developed to handle motion sensors and map landmarks, it works efficiently with solely visual information [24]. In this case, a map of the environment is built, and, at the same time, it is used to deduce the relative pose which is represented using probabilistic models. Several solutions to SLAM involve finding an appropriate representation for the observation model and motion model while preserving an efficient and consistent runtime. Most methods use additive Gaussian noise to handle the uncertainty which is imposed using the extended Kalman filter (EKF) to solve the SLAM problem [34]. In cases where visual features are used, EKF may fail to estimate the trajectory accurately due to the significant uncertainties that appear in large loops [13]. Additionally, runtime and used resources grow constantly for large environments. Later works tried mainly to handle scalability issues.

A remarkable improvement of SLAM is the FastSLAM approach [12] which aims at greater scalability. It uses recursive Monte Carlo sampling to directly represent the non-linear process model. Although the state-space dimensions are reduced when the Rao–Blackwellisation approach is used [39], the method remains not scalable to large autonomy. In the context of long trajectories, several solutions have been proposed to handle relative map representations, such as [22,24,40,41]. In particular, these involve breaking the estimation into smaller mapping regions, called sub-maps, and then computing individual solutions for each sub-map. In the same manner, hierarchical SLAM [13] divides the map into two levels—a lower level that is composed of a set of a sequence of local maps of limited size and an upper level that handles the relative relations between local maps, which are maintained using a stochastic approach. Although these solutions perform well in large environments, sub-mapping is not efficient for raster scanning/motion as this will cause very frequent sub-maps switches. Also, there are some issues in defining the size, overlapping, and the fusion of sub-maps.

In all reviewed SLAM methods, in case of using pure visual information, the measurement noise (such for relative motion estimation) is modeled by a diagonal covariance matrix with equal variances that are set empirically [14]. This modeling leads to the production of uncorrelated measurement error among dimensions. However, the estimated pose should have an associated full degrees of freedom (DOF) uncertainty. Although several works exist in the literature that studied the uncertainty of 3D reconstructed points based on their distance from the camera and the baseline distance between frames, such as [19,42], or the matching error propagation in 3D, such as [9], the effect of the relative motion parameters on the uncertainty of the pose estimation has not been taken into account.

From another perspective, visual odometry methods use structure from motion (SfM) methodology to estimate the relative motion [18]. Based on multiple view geometry fundamentals [43], an approximate relative pose can be estimated. This is followed by a BA procedure to minimize re-projection errors, which yields an improvement in the estimated structure. Fast and efficient BA approaches are proposed to be able to handle a large number of images [44]. However, in the case of longtime navigation, the number of images increases constantly and prevents the application of global BA if real-time performance is needed. Hence, several local BA approaches have been proposed to handle this problem. In local BA, a sliding window copes with motion and select a fixed number of frames to be considered for BA [10]. This approach does not suit the raster scans commonly used in surveys, since the last *n* frames to the current frame are not necessarily the closest. Another local approach is relative BA, proposed in [45]. Here, the map is represented as a Riemannian manifold-based graph with edges representing the potential connections between frames. The method selects the part of the graph where the BA will be applied by forming two regions—an active region that contains the frames with an average re-projection error changes by more than a threshold, and a static region that contains the frames that have common measurements with frames in the active region. When performing BA, the static region frames are fixed, whereas active region frames are optimized. The main problem with this method is that distances between frames are deterministic, whereas the uncertainty is not considered when computing inter-frame distances.

In the context of underwater robotics, SLAM solutions based on active sensors, such as DVL, the Inertial Navigation Unit (INU) and Side Scan Sonars (SSS) have mostly been proposed [46,47]. An early attempt to use a vision system was proposed in [48], where a fusion is performed between sonar and visual information, and a Lucas Kanade feature tracking is applied to the image stream—it is used to only extract robot’s bearing observation which does not generalize to free motion. A more general solution was proposed in [49], in which the ego-motion is estimated by finding the rigid transformation between two point clouds which are generated using a stereo system at two time intervals. The relative motion is then integrated with SLAM which uses an SSS as well. Works relying solely on visual sensors are surprisingly rare; noticeably, they use the same terrestrial SLAM techniques as those reviewed above [50]. The majority of these methods rely on stereo vision to estimate metric trajectory [9,28,51].

## 3. Hardware Platform

Throughout our hardware design and implementation, we were committed to a low-cost solution. Thanks to the latest developments of single-board computers, power-efficient systems equipped with a high-performance multi-core CPUs, and most modern peripheral networking interfaces are available in the size of a credit card. Being increasingly available and cheap, we chose the popular Raspberry Pi (RPi) [52] (a credit card-sized ARM architecture-based computer with 1.2 GHz 64-bit quad-core CPU and 1 GB of memory, running Rasbain, a Linux-based operating system. We used RPi version 3 in this project) as the main processing unit of our platform. This allowed most image processing and computer vision techniques to be run smoothly. As already mentioned, we designed and implemented two configurations of our system that we present in the following text.

### 3.1. ROV-Attached Trifocal System

The design here is based on the BlueROV2 from BlueRobotics [2], Figure 2 shows the full system design and implementation. The ROV is equipped with six thrusters (four vectored and two vertical), controlled by a Pixhawk autopilot [53] which allows 4 DoF navigation to be performed—all but pitch and yaw. The ROV is operated from a surface computer (laptop) that also receives the live video feedback. We used 4×1500 lumens diffuse led torches for lighting oriented at a tilt of $135^{\circ }$.

**Figure 3:**
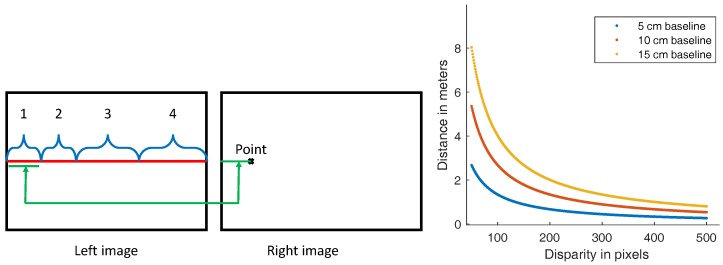
Illustration of stereo disparity ranges (**left**): (1) impossible due to stereo constraint; (2) impossible in deep underwater imaging due to light fading at far distances; (3) possible disparity; (4) the point is very close, so it becomes overexposed, undetectable, or out of focus. At (**right**), the disparity evaluation in pixels as a function of distance (in meters) to the camera for the 3 available baseline distances.

**Figure 4:**
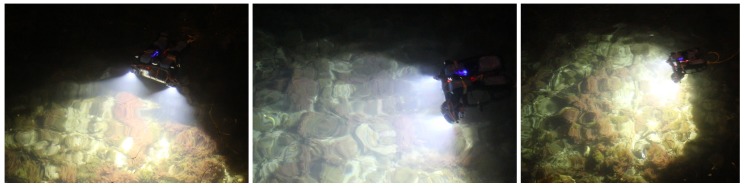
The built trifocal Remotely Operated underwater Vehicle (ROV)-attached system in action.

A cylindrical enclosure (34 × 15 cm) is attached to the front side of the ROV, as shown in Figure 2. It hosts the designed trifocal system, which is composed of three RPi computers; each is connected to one camera module (Sony IMX219 8M Pixel $1/4^{\prime \prime }$ CMOS Image Sensor, 3 mm focal length, f/2 aperture). Using the trifocal system allows three stereo pairs with different baseline distances (set to 5, 10 and 15 cm in our implementation) to be present, which helps to handle image acquisition at different distances. Figure 3 (right) shows the range of each baseline distance. Here, we can deduce that a short baseline is preferred in close-range image acquisition. For instance, with the used configurations, it is difficult to get closer than 80, 53 and 26 cm to the scene using 15, 10 and 5 cm baseline distances, respectively. From another perspective, small baseline distances are less accurate for larger distances. We note here that the visibility limit underwater (≈5 m using our lighting system) is much smaller than the stereo range whatever the used baseline distance. The cameras are synchronized using a hardware trigger connected to the general-purpose input/output (GPIO) interface of the RPi computers. The latter are finally connected to an Ethernet switch that is connected to the surface computer. Figure 4 shows the ROV-attached trifocal system in action.

### 3.2. Handheld Stereo System

**Figure 5:**
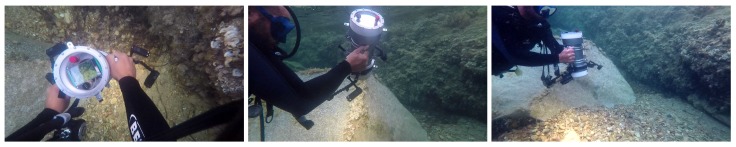
The built handheld stereo system in action.

An illustration of the built handheld system is shown in Figure 1. It is composed of two RPi computers; each is connected to one camera module to form a stereo pair. The cameras are synchronized using a hardware trigger in the same manner as the previous system. Both RPi computers are connected through Ethernet to the surface. A high contrast monitor is embedded in the same enclosure and is visible from outside (see Figure 5). The monitor is attached to one of the RPi computers and shows real-time preview and diverse information, such as image quality, storage, and connections.

In both designed systems, the embedded computers are responsible for image acquisition. The captured stereo images are first partially processed on the fly to provide image quality information, as will be detailed in Section 4. Images are then transferred to a central computer which handles the computation of the ego-motion that the system undergoes. This will be detailed in Section 5. We note that our implementation assumed calibrated stereo pairs. Therefore, we employed a traditional but efficient approach that uses an underwater target of chessboard pattern and the camera calibration toolbox in OpenCV [3]. The procedure was performed offline before the mission. After observing stable extrinsic parameters of two trials, we did not perform any further recalibration.

## 4. Image Acquisition and Quality Estimation

Since underwater images do not tend to be in the best condition, a failing scenario in computing the ego-motion is expected and has to be considered. Here, we could encounter two cases. First, when there is a degenerated configuration that causes a failure to estimate the relative motion, this can be due to poor image quality (blurred, dark or overexposed), lack of textured areas or large camera displacements. This may raise ill-posed problems at several stages. Second, imprecise estimation of the relative motion due to poorly distributed feature points or the dominant presence of outliers in the estimation procedure may occur. While a mathematical analysis can identify the first failure case, the detection of the second case is not trivial. Nevertheless, small errors can be corrected later using the BA procedure.

A real-time image quality estimation provides two benefits: first, it can alert the visual odometry process of having poor image quality. Two reactions can be taken in this case, either pausing the process until the taken image quality goes above a certain threshold or producing position estimation based on previous poses and speed. We went for the first case while leaving the second for further development in the future. Second, the image quality indicator provides direct information to the operator to avoid it going too fast in case of a blur or changing the distance to the captured scene when it is under or over-exposed.

To estimate the image sharpness, we rely on an image gradient measure to detect the high frequencies often associated with sharp images. Thus, we used Sobel kernel-based filtering which computes the gradient with a smoothing effect. This removes the effect of dust commonly present in underwater imaging. Given an image, I, we start by computing the image gradient magnitude, G, as

(1) $$G=\sqrt{{\left(SK^{⊤}*I\right)}^{2}+{\left(KS^{⊤}*I\right)}^{2}},$$

where

$S={\left[1\;2\;1\right]}^{⊤}$
$K={\left[-1\;0\;1\right]}^{⊤}$
* is a convolution operator.

We consider our sharpness measure to be the mean value of G. A threshold can be easily learned from images by solving a simple linear regression problem. First, we record the number of matched feature points per image versus the sharpness indicator. Then, by fixing the minimum number of matched feature points needed to estimate the ego-motion correctly, we can compute the minimum sharpness indicator threshold (in our experiments, we fixed the number of matches to 100 matches; the obtained threshold was ≈20). It is worth noting that several assumptions used in our work, including this measure, do not hold for terrestrial imaging scenarios. In particular, the seabed texture guarantees a minimum sharpness even in object-free scenes.

From another perspective, good scene lighting yields better images, so it influences the accuracy of odometry estimation. Similar to the image sharpness indicator, an image lightness indicator can be integrated into the odometry process as well as helping the operator to take proper actions. To estimate the lightness indicator, we convert the captured images to the CIE-XYZ color space and then to the CIE-LAB color space. We consider the lightness indicator to be the mean value of the lightness channel *L* (using a percentile-based measure, such as the median, is more representative but it takes around 22 times longer to compute than the mean). The threshold is computed in the same way as for the sharpness.

Both indicators are computed using a sub-sampled image without interpolation. This allows the processing time to be decreased by 80%, with an average time of 60 ms on a single RPi computer, while keeping the accuracy above 95% compared to using the full resolution images.

## 5. Visual Odometry

After computing and displaying the image quality measures, the images are transferred over the network to the surface computer (average laptop computer). This computer is responsible for hosting the visual odometry process, which will be explained in this section. We begin by introducing the used stereo matching approach, and then we present the ego-motion estimation. Finally, we explain the semi-global BA approach.

### 5.1. Speeded Up Stereo Matching

**Figure 6:**
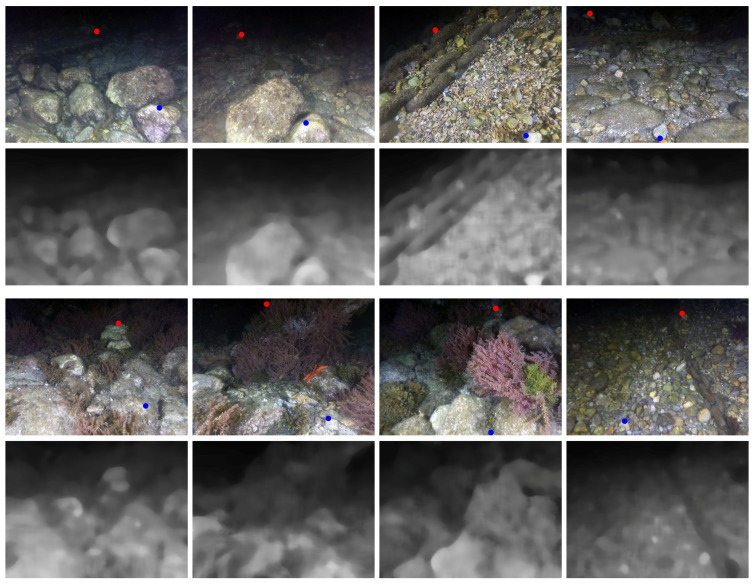
Examples of underwater images taken with our system and the computed rough depth using only the luminance channel. The rough depth was used later to speed up the stereo matching procedure. The red dots show the minimum detectable disparity (≈130 pixels in 10 cm baseline setup), while the blue dots show the maximum disparity (≈450 pixels in 10 cm baseline setup). See Figure 3 for corresponding distances and conversion to other baselines.

Matching feature points between stereo images is essential for the estimation of ego-motion. As the alignment of the two cameras is not perfect, we start by calibrating the camera pair. Hence, for a given point on the right image, we are able to compute the epipolar line containing the corresponding point in the left image. However, based on the known fixed geometry, the corresponding point position is constrained by a positive disparity. Moreover, given that, in deep water, the only light source is the one attached to our system, the farthest distance that feature points can be detected is limited (see Figure 6 for illustration). This means that there is a minimum disparity value that is greater than zero; the red dots in Figure 6 refer to the minimum disparity, for instance. It was at least 130 pixels for the 10 cm baseline stereo pair. Furthermore, when going too close to the scene, parts of the image will become overexposed, undetectable, or out of focus. Similar to the previous case, this imposes a limited maximum disparity. Figure 3 illustrates the constraints mentioned above by dividing the epipolar line into four ranges, in which only one was an acceptable disparity in our context. This range can be directly identified by learning from a set of captured images (oriented at $30^{{}^{\circ}}$ for better coverage).

**Figure 7:**
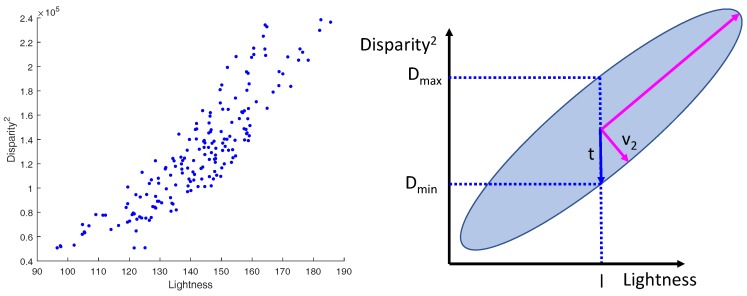
Disparity vs. lightness relationship for a subset of matched points. **Left**: local average pixel lightness vs. squared disparity. **Right**: an illustration of disparity tolerance, *t*, for a given lightness, *l*.

In our approach, we propose to constrain the so-defined acceptable disparity range further, which corresponds to the third range in Figure 3(left). Given the used lighting system, we can assume a light diffuse reflection model where the light reflects equally in all directions. Based on the inverse-square law that relates light intensity over distance, image pixels intensities are roughly proportional to their squared disparities. Based on such an assumption, we can use the pixel intensity to constrain the disparity and hence, limit the range of searching for a correspondence. To do so, we used a dataset of rectified stereo images. For each image pair, we performed feature point matching. Moreover, for each matching pair of points $\left(x_{i},y_{i}\right)$ and $\left(x_{i}^{\prime },y_{i}^{\prime }\right)$, *x* being the coordinate in the horizontal axis, we computed the squared disparity, $d_{i}^{2}={\left(x_{i}-x_{i}^{\prime }\right)}^{2}$. Next, we associated each $d_{i}^{2}$ to the mean lightness value, denoted ${\bar{l}}_{x_{i},y_{i}}$, of a window centered at the given point computed from the lightness channel, *L*, in the CIE-LAB color space. We assigned a large window size of (≈15) to compensate for using the Harris operator that promotes local minimum intensity pixels as salient feature points. Several examples of the computed rough depth maps are shown in Figure 6. The computed $\left({\bar{l}}_{x_{i},y_{i}},d_{i}^{2}\right)$ pair shows the linear relationship between the squared disparity and the average lightness. A subset of such pairs is plotted in Figure 7(left).

In addition to finding the linear relationship between both variables, it was also necessary to capture the covariance that represents how rough our approximation is. More specifically, given the relation shown in Figure 7, we aim to define a tolerance, *t*, associated with the disparity as a function of the lightness, *l*. In our method, we rely on the Principal Component Analysis (PCA) technique to obtain this information. In detail, for a given lightness, *l*, we first compute the corresponding squared disparity, $d^{2}$, using a linear regression approach as follows:

(2) $$d^{2}=-\alpha l-\beta$$

where

(3) $$\begin{array}{cc} \alpha & =\frac{Cov(L,D^{2})}{Var(L)} \end{array}$$

(4) $$\begin{array}{cc} \beta & =\bar{l}-\alpha \bar{d^{2}}, \end{array}$$

where *D* and *L* , both vectors of n×1 with *n* being the data size, are the disparity and the lightness training vectors, respectively, and $\bar{d}$ and $\bar{l}$ are their respective means. Second, let $V_{2}={\left[v_{2,x}\;v_{2,y}\right]}^{⊤}$ be the computed eigenvector that corresponds to the smallest eigenvalue, $\lambda_{2}$, of the n×2 matrix $\left[L\;D^{2}\right]$. Based on the illustration shown in Figure 7 (right), the tolerance, *t*, associated with $d^{2}$ can be written as:

(5) $$t=\sqrt{\lambda_{2}^{2}\left(\frac{v_{2,x}^{2}}{v_{2,y}^{2}}+1\right)}.$$

By considering a normal error distribution of the estimated rough depth and based on the fact that *t* is related to the variance of $D^{2}$, we define the effective disparity range as

(6) $$d\pm \gamma \sqrt[{4}]{t},$$

where γ represents the number of standard deviations. It is trivial that γ is a trade-off between the runtime and the probability of having point correspondences within the chosen tolerance range. We set γ=2 which that means there is 95% probability of covering the data. In practice, this translates to less than 100 pixels, which is a significant reduction of the searching range (the used camera has a resolution of 3280×2464, or 1640×1232 in a faster binned-mode).

**Figure 8:**
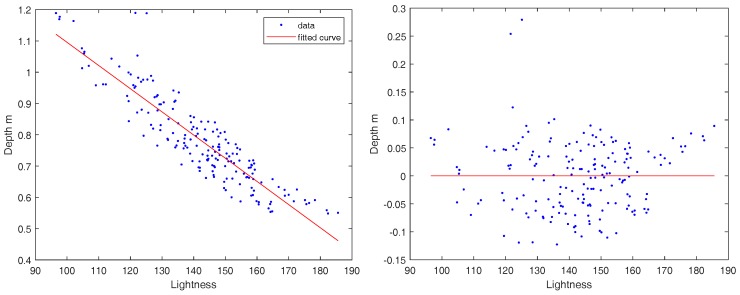
Lightness vs. true depth relationship for a subset of matched points (the same as that used in Figure 7). **Left**: local average pixel lightness vs. true depth (10 cm baseline); the red line represents the lightness to depth transformation—it is deduced from Equation (2). **Right**: the residuals of the depth estimation from lightness vs. true depth.

The proposed methodology deals with errors in the rough depth estimation. For example, the rock, which appears in the first image of the second row in Figure 6, is farther away from where it is estimated in the rough depth map. This is due, generally, to variable surface reflectance among underwater objects or the angle of light incidence. We note that a general indication of the rough depth estimation quality is the eigenvalue (a smaller value means better depth estimation) that corresponds to the eigenvector, $V_{2}$, as it represents a deviation in the lightness value from the linear relationship given in Equation (2). An illustration of a true depth (computed from disparity) vs. the depth estimated from the lightness is shown in Figure 8 (left). The residuals of this estimation are illustrated in Figure 8 (right). We reiterate that the range defined in Equation (6) leaves a sufficient margin to account for the deviation from the true value.

### 5.2. Initial Ego-Motion Estimation

**Figure 9:**
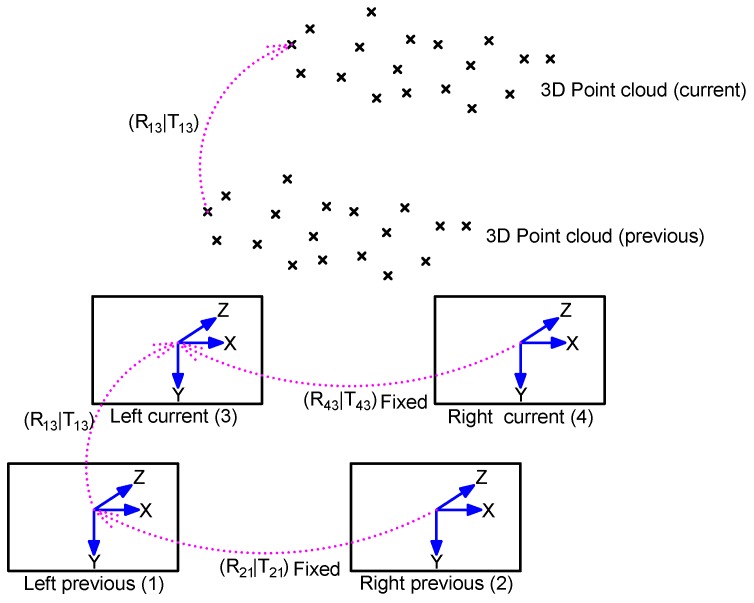
Image quadruplet: the current (left and right) and previous (left and right) frames are used to compute two 3D point clouds. The transformation, $\left[R_{13}|T_{13}\right]$, between the two points clouds is equal to the relative motion between the two camera positions.

An initial ego-motion is calculated every time a new image pair is captured. Let $\left(f_{1},f_{2},f_{3},f_{4}\right)$ denote the previous left, previous right, current left, and current right frames, respectively (see Figure 9 for illustration). We consider here that the relative positions of the previous left frame to the current left frame ($f_{1}\to f_{3}$) represent the system motion. The pipeline of the ego-motion estimation proceeds is as follows:

1. Feature point detection of $f_{1}$ using the Harris-based Shi–Tomasi method [26].
2. Perform feature point matching using the patch descriptor (11×11, as advised in [8]), and the normalized sum of squared differences as a distance measure for the frames ($f_{1}$, $f_{2}$). Given the camera calibration parameters, the search range across the epipolar lines is reduced using the analysis presented in Section 5.1.
3. The feature points detected in $f_{1}$ are tracked in $f_{3}$ using the Pyramidal Lucas–Kanade (LK) method [54].
4. The fundamental matrix is computed for the frames ($f_{1}$, $f_{3}$) using the normalized eight point method with RANSAC as described in [43]. The matrix is used to reject the tracking outliers. This step is optional—although it improves the accuracy slightly, more computation time adds up.
5. Repeat Step 2 for frames ($f_{3}$, $f_{4}$) using the tracked feature points found in Step 3.
6. Compute two 3D point clouds using triangulation for the matched feature points in frames ($f_{1}$, $f_{2}$) and ($f_{3}$, $f_{4}$) respectively. We note that the correspondence between the two point clouds is known.
7. Compute the relative transformation between the two 3D point clouds, which represents the ego-motion that the ROV undergoes (to be explained in the following text).

We note that starting from the second run of the procedure, Step 1 is appended so that the detected feature points are first compared against those of the previous estimation (using a truncated resolution of 0.1 pixel). This yields two groups of points: new detections and the points that have been already processed from the previous run. Their correspondence is already established within the $f_{2}$ frame, and their 3D position is computed. Hence, Steps 2–6 will only be computed for new detections.

The choice of using the LK approach is justified by the relatively slow scene change over time, which is reasonably correct due to system mass and smooth motion underwater. Since the LK method employs a closed-form formulation to measure the optical flow, it remains faster than a patch matching scheme. However, it does not suit stereo matching due to the large disparity between corresponding points (up to several hundreds of pixels, as seen earlier).

As there is no scaling problem between the two 3D point clouds, the relative transformation can be expressed as a 3×3 rotation matrix *R* and a 3×1 translation vector, *T*, namely $\left[R_{13}|T_{13}\right]$ (see Figure 9). The method to compute this transformation is presented in the following text. Let *P* and $P^{\prime }$ be the point clouds associated with the image pairs $\left(f_{1},f_{2}\right)$ and $\left(f_{3},f_{4}\right)$, respectively. Let $p_{i}\in P$ and $p_{i}^{\prime }\in P^{\prime }$ be two homologous points (correspondence relationship established in Step 3). We have:

(7) $$p_{i}^{\prime }=R_{13}\;p_{i}+T_{13}.$$

We seek a transformation the minimizes the error, *r*, the sum of squared residuals:

(8) $$r=\sum_{i=1}^{n}{∥R_{13}\;p_{i}+T_{13}-p_{i}^{\prime }∥}^{2}.$$

To solve this problem, we follow the method proposed in [55]. Briefly, a 3×3 matrix *C* is formed as

(9) $$C=\sum_{i=1}^{n}\left(p_{i}-\bar{p}\right){\left(p_{i}^{\prime }-{\bar{p}}^{\prime }\right)}^{⊤},$$

where $\bar{p}$ and ${\bar{p}}^{\prime }$ are the centers of mass of the 3D point sets, *P* and $P^{\prime }$, respectively. Given $C=USV^{⊤}$, the singular value decomposition (SVD) of the matrix, *C*, the final transformation is computed as

(10) $$\begin{array}{cc} R_{13} & =VU^{⊤} \end{array}$$

(11) $$\begin{array}{cc} T_{13} & =-R_{13}\bar{p}+{\bar{p}}^{\prime }. \end{array}$$

This solution could potentially return reflected rotations, where $det(R_{13})=-1$. This can be corrected by multiplying the third column of $R_{13}$ by −1.

Once the image pair, $\left(f_{3},f_{4}\right)$, is expressed in the reference system of the image pair, $\left(f_{1},f_{2}\right)$, the 3D points can be recalculated using the four observations that we have for each point. A set of verifications are then performed to minimize the pairing errors (verification of the epipolar line, the consistency of the y-parallax, and re-projection residuals). Once validated, this initial ego-motion estimation is used in the BA procedure that will be described later.

### 5.3. Uncertainty in Visual Odometery

**Figure 10:**
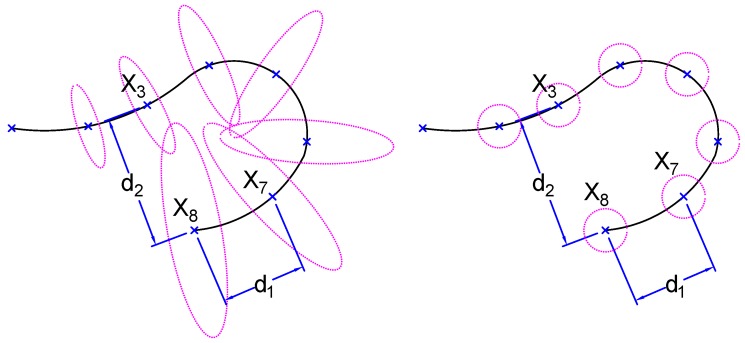
Example of a trajectory with uncertainty modeled by the full covariance matrix (**left**). The distance, $d_{2}$, is statistically estimated to be smaller than $d_{1}$. In contrast, it is the inverse when the noise is modeled with equal variances (**right**).

As shown in the literature review, relative visual odometry represents a good solution for long-term autonomy. This kind of approach deals with a selected region of the map at a time. The aim is to reduce the optimization runtime for a new pose. In detail, given a set of frames resulting from camera trajectory. For a new frame at time, *t*, the pose is estimated w.r.t. frame t−1. Here, a relative approach would perform a selection of frames within a certain distance. These frames are assumed to have the largest potential overlap with the current frame. Using these frames, BA is performed to optimize the trajectory. As we have seen earlier, most of the proposed methods assume equal and uncorrelated Gaussian noise for all axes. This is illustrated in Figure 10 (right). In this case, when searching for the nearest frames to be included in the optimization process, the distance, $d_{2}$, is larger than $d_{1}$, both geometrically and statistically. However, having a full covariance representation of the pose, for instance, as shown in Figure 10 (left), the Euclidean distance measure is no more appropriate. Here, any divergence measure would estimate $d_{2}$ to be smaller than $d_{1}$, which is more realistic. Since the visual odometry approach suffers from drifting, it is essential to consider an efficient uncertainty measure to represent and determine adjacent frames.

Like any visual odometry estimation, the estimated trajectory using the method mentioned in the previous section is exposed to a computational error, which translates to some uncertainty that grows over time. A global BA may handle this error accumulation; however, it is time-consuming. From another side, a local BA is a tradeoff for accuracy and runtime. The selection of *n* closest frames is made using the standard Euclidean distance. Loop closure may occur when overlapping with already visited areas which, in return, enhances the accuracy. This approach remains valid as soon as the uncertainty is equal for all estimated variables. However, as the uncertainty varies, the selection of the closest frames based on the Euclidean distance is not suitable. In the following text, we prove that it is the case for any visual odometry method. Also, we provide a formal definition of the uncertainty associated with ego-motion estimation.

Most visual odometry and 3D reconstruction methods rely on matched feature points to estimate the relative motion between two frames. The error in the matched features is resulting from several accumulated errors. These errors are due, non-exclusively, to the following reasons: optical distortion modeling, the discretization of 3D points when projected to image pixels, motion blur, depth of field blur, internal camera noise, salient points detection, and matching. By performing image undistortion, and constraining the point matching with the fundamental matrix, the accumulation of the errors mentioned above can be approximated with a Gaussian distribution. This is implicitly considered in most computer vision fundamentals. Based on this assumption, we can prove that the error distribution of the estimated relative pose is unequal among dimensions. Indeed, it can be fitted to a multivariate Gaussian whose covariance matrix has unequal Eigenvalues, as we will see later.

To better demonstrate this idea, we will take the traditional example of computing the relative pose by means of the fundamental matrix (the results of this analysis also hold for our method, which will be considered in Section 5.4). Formally, a pair of matched points, $m↔m^{\prime }$, between two frames, can be represented by a multivariate Gaussian distribution $N\left(m,\Sigma \right)↔N\left(m^{\prime },\Sigma \right)$, where $\Sigma =diag(\sigma^{2},\sigma^{2})$. The pose estimation procedure relies on the fundamental matrix that satisfies $m^{\prime }\;F\;m=0$. Writing $m={\left[x\;\;y\;\;1\right]}^{⊤}$ and $m^{\prime }={\left[x^{\prime }\;\;y^{\prime }\;\;1\right]}^{⊤}$ in homogeneous coordinates, the fundamental matrix constraint for this pair of points can be written as

(12) $$x^{\prime }xf_{11}+x^{\prime }yf_{12}+x^{\prime }f_{13}+y^{\prime }xf_{21}+y^{\prime }yf_{22}+y^{\prime }f_{23}+xf_{31}+yf_{32}+f_{33}=0,$$

where $f_{ij}$ is the element at row *i* and column *j* of F. To show the estimated pose error distribution, we consider one configuration example, the identity camera intrinsic matrix, K=diag(111). Let us now take the case of pure translational motion between the two camera frames, $T={\left[T_{X}\;T_{Y}\;T_{Z}\right]}^{⊤}$, and $\theta ={\left[\theta_{x}\;\theta_{y}\;\theta_{z}\right]}^{⊤}={\left[0\;0\;0\right]}^{⊤}$, where T and θ are the translation and rotation vectors respectively. The fundamental matrix, in this case, is given as a skew-symmetric matrix of T, denoted ${\left[T\right]}_{\times }$. In this case, Equation (12) is simplified to

(13) $$-x^{\prime }yT_{Z}+x^{\prime }T_{Y}+y^{\prime }xT_{Z}-y^{\prime }T_{X}-xT_{Y}+yT_{X}=0.$$

By using enough matched points, we can recover the translation vector, T, by solving a linear system. However, the Gaussian error associated with $x,y,x^{\prime }$ and $y^{\prime }$ will propagate equally to variables $T_{X}$ and $T_{Y}$, with a variance equal to $2\sigma^{2}$. In contrast to $T_{Z}$ where the error distribution is different due to the product of two variables, each follows a Gaussian distribution. In addition to not being Gaussian-distributed in general cases, their product’s variance is approximate (there is no analytical solution to find the variance of the product of two Gaussian distributed variables). The product of two Gaussian distributed variables follows a normal product distribution; it has been proven that it tends towards a normal distribution when μ/σ is large enough, which is not the case here. Alternatives include numerical integration, Monte Carlo simulation and analytical approximation. The given formula results from the latter case $\sigma^{2}\left(x^{2}+y^{\prime 2}+x^{\prime 2}+y^{2}\right)$, which largely exceeds the error variance associated with $T_{X}$ and $T_{Y}$.

Moreover, due to the usage of least square approach through an SVD decomposition, as in our method, or two consecutive SVDs (used for fundamental matrix computation and essential matrix decomposition) in traditional visual odometry, the error distributions associated with recovered pose parameters are correlated (even though the observations are uncorrelated), as explained in [56]; this is also demonstrated experimentally in the next sub-section. Overall, this leads to having the estimated pose follow a Gaussian distribution with a full DOF covariance matrix (within the symmetric positive semi-definite constraint).

### 5.4. Pose Uncertainty Modeling and Learning

**Figure 11:**
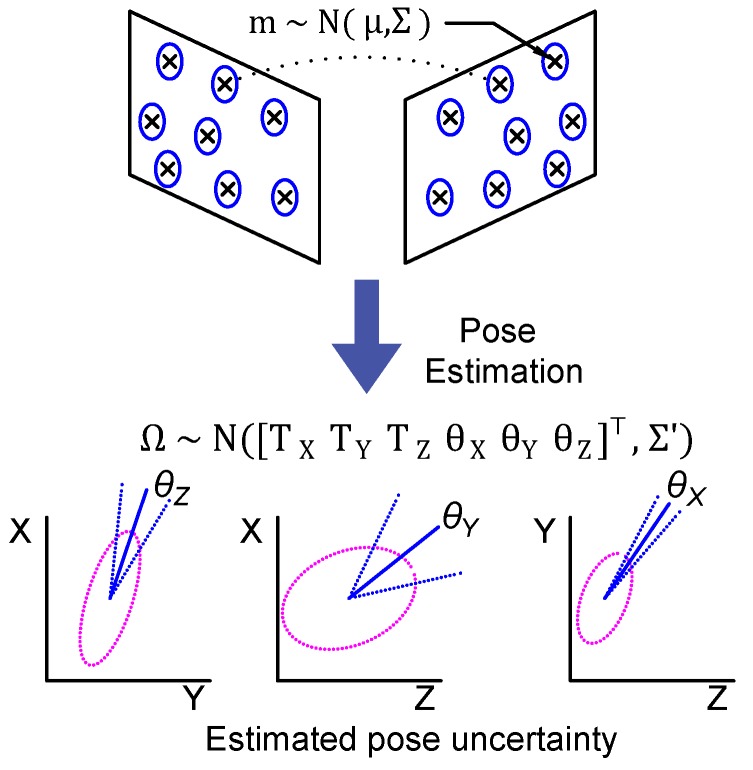
Illustration of error propagation through the pose estimation procedure. The estimated pose uncertainty is shown for each of the six DOF. A full error covariance matrix could result from uncorrelated error distribution of matched 2D feature points.

Pose uncertainty is difficult to estimate analytically. This is due to the complexity of the pose estimation procedure and the number of variables involved. In particular, the noise propagation through SVD decomposition cannot be analytically modeled. Instead, inspired by the unscented Kalman filter approach [57], we proceed similarly by simulating the noisy input and trying to characterize the output error distribution in this case. This process is illustrated in Figure 11. In our work, we propose to learn the error distribution based on finite but numerous pose samples. This is done using a neural network approach which fits well with our problem as it produces a soft output.

Two factors play a role in the estimated pose uncertainty. First, the motion, $\Omega ={\left[T\;\theta \right]}^{⊤}$, between the two frames is expressed by a translation T and a rotation θ, which is explained in the previous section. Second, is the 3D location of the matched feature points. Although their locations are not computed explicitly in our method, their distances from the camera affect the computation accuracy. In particular, the further the points are from the camera the less accurate the estimated pose is. This is because close points yield larger 2D projection disparity which becomes less sensitive to discretization error. For instance, in a pure translation motion, if all matched points are within the blind zone of the vision system (produce zero-pixel disparity after discretization), the estimated motion will be equal to zero. This problem can be solved with points closer to the camera. Both mentioned factors are correlated to some extent. For instance, given some points in 3D (*n* > 7), the estimated pose accuracy is a function of their depth, but also of the baseline distance and the angle between the two optical centers of the cameras [43] (p. 323). Hence, considering one factor is sufficient. In our work, we consider the motion as a base to predict the uncertainty.

Formally, given a motion vector, $\Omega ={\left[T\;\theta \right]}^{⊤}$, ideally, we seek to find the covariance matrix that expresses the associated error distribution. Being positive semi-definitive (PSD), an n×n covariance matrix has $(n^{2}+n)/2$ unique entries. Having n=6, in our case, yields 21 DOF, of which six are variances. However, learning this number of parameters freely violates the PSD constraint. Whereas finding the nearest PSD, in this case, distorts the diagonal elements largely because of being mush less. At the same time, we found, experimentally, that the covariance between T and θ variables is relatively small compared to that of intra T and intra θ. Thus, we consider the estimation of two distinct covariance matrices, $\Sigma_{T}$ and $\Sigma_{\theta }$. So, in total, we have 12 parameters to learn, of which six are the variances.

For the aim of learning $\Sigma_{T}$ and $\Sigma_{\theta }$, we created a simulation of the pose estimation procedure. For a fixed well-distributed 3D points $X_{i}\in R^{3}:i=1.8$, we simulated two cameras (to form a stereo pair) with known intrinsic and extrinsic values. The points were projected according to both cameras’ 2D image points. A motion vector, Ω, was applied to the cameras. Then, the 3D points were projected again. All projected points were then disturbed with random Gaussian noise. Next, the ego-motion was estimated by applying the method proposed in Section 5.2 on the disturbed points. Let $\tilde{\Omega }={\left[\tilde{T}\;\tilde{\theta }\right]}^{⊤}$ be the estimated motion. Repeating the same procedure (with the same motion Ω) produced a set of motion vectors which represented a point cloud of poses around the real one. Next, we computed the covariance matrices, $\Sigma_{T}$ and $\Sigma_{\theta }$, of the resulting motion vectors to obtain the uncertainty associated with the given motion, Ω. Furthermore, this procedure was repeated for a large number of motion vectors that covered a wide ranges of its six composing variables (in the performed simulation, we use the range [0−1] with a step size of 0.25 for the translation for each of the 3 dimensions. For rotations, we used the range [0−π/2.5] with a step of π/10. This raised up to 15,625 training samples).

At this stage, having produced the training data by means of motion vectors and the corresponding covariance matrices, we proceeded to build a system to learn the established correspondences (motion ⇔ uncertainty), so that, in the case of new motion, we would be able to predict the uncertainty. Neural networks offer this soft output by nature, which is the reason why we adopted this learning method. In our experiments, we found that a simple neural network with a single hidden layer [58] was sufficient to fit the data well. The input layer had six nodes that corresponded to the motion vector. The output layer had 12 nodes which corresponded to the unique entries in $\Sigma_{T}$ and $\Sigma_{\theta }$. Thus, we formed our output vector as

(14) $$O={\left[\Sigma_{T}^{11}\;\Sigma_{T}^{22}\;\Sigma_{T}^{33}\;\Sigma_{T}^{12}\;\Sigma_{T}^{13}\;\Sigma_{T}^{23}\;\Sigma_{\theta }^{11}\;\Sigma_{\theta }^{22}\;\Sigma_{\theta }^{33}\;\Sigma_{\theta }^{12}\;\Sigma_{\theta }^{13}\;\Sigma_{\theta }^{23}\right]}^{⊤},$$

where $\Sigma_{\cdot }^{ij}$ is the element of row *i* and column *j* of a covariance matrix. In the learning phase, we used the Levenberg–Marquardt backpropagation which is a gradient-descent based approach, as described in [59]. Further, by using the mean-squared error as a cost function, we were able to achieve around a training error rate of 3%. The obtained parameters were rearranged into two symmetric matrices. In practice, the obtained matrix is not necessarily PSD. We proceeded to find the closest PSD as $Q\Lambda_{+}Q^{-1}$, where *Q* is the eigenvector matrix of the estimated covariance matrix, and $\Lambda_{+}$ is the diagonal matrix of eigenvalues, in which negative values are set to zero.

To validate the training phase, the procedure to generate the training set was repeated but using different values of motion vectors. The validation of this test set using the trained neural network showed an accuracy of 87.6% and a standard deviation of 6.1, which is reasonably acceptable in this context.

### 5.5. Semi-Global Bundle Adjustment

After initiating the visual odometry, the relative pose estimation at each frame is maintained within a table that contains all poses’ related information (18 parameters per pose, in which 6 for the position, and 12 for two covariance matrices). At any time, it is possible to identify the poses in the neighborhood of the current pose being estimated to find potential overlaps to consider while performing BA. Since we were dealing with a statistical representation of the observations, a divergence measure had to be considered. Here, we chose the Bhattacharyya distance as suitable for our problem (modified metric variation could be also used [60]). Formally, the distance between the two poses, $\left\{\Omega^{1},\Sigma_{T}^{1},\Sigma_{\theta }^{1}\right\}$ and $\left\{\Omega^{2},\Sigma_{T}^{2},\Sigma_{\theta }^{2}\right\}$, is given as:

(15) $$D=\frac{1}{8}{\left(\Omega^{1}-\Omega^{2}\right)}^{⊤}\Sigma^{-1}\left(\Omega^{1}-\Omega^{2}\right)+\frac{1}{2}\ln\left(\frac{\det\Sigma }{\sqrt{\det\Sigma^{1}+\det\Sigma^{2}}}\right),$$

where

(16) $$\Sigma^{\cdot }=\left[\begin{array}{cc} \Sigma_{T}^{\cdot } & 0\\ 0 & \Sigma_{\theta }^{\cdot } \end{array}\right],\;\;\Sigma =\frac{\Sigma^{1}+\Sigma^{2}}{2}.$$

Having selected the set of frames, F, in the neighborhood of the current pose statistically, we performed BA as follows; First, we divided F into two subsets similar to [45]. The first subset, $F_{d}$, contained the current and previous frames in time, whereas the other subset $F_{s}$ contained the remaining frames, mostly resulting from overlap with an already scanned area. Second, BA was performed on both subsets. However, the pose parameters related to $F_{s}$ were masked as static, so they were not optimized, in contrast to $F_{d}$. This strategy was necessary to reduce the number of variables to optimize.

After determining the error distribution arising with a new pose, it has to be compounded with the error propagated from the previous pose. Similar to SLAM approaches, we propose to use a *Kalman filter* like gain which allows controllable error fusion and propagation. Given an accumulated previous pose estimation, defined as $\left\{\Omega^{p},\Sigma_{T}^{p},\Sigma_{\theta }^{p}\right\}$, and a current one, $\left\{\Omega^{c},\Sigma_{T}^{c},\Sigma_{\theta }^{c}\right\}$, an updated current pose, $\left\{\Omega^{u},\Sigma_{T}^{u},\Sigma_{\theta }^{u}\right\}$, is calculated as:

(17) $$\begin{array}{cc} \Omega^{u} & =\Omega^{c} \end{array}$$

(18) $$\begin{array}{cc} \Sigma_{\theta }^{u} & =\left(I-\Sigma_{\theta }^{p}{\left(\Sigma_{\theta }^{p}+\Sigma_{\theta }^{c}\right)}^{-1}\right)\Sigma_{\theta }^{p} \end{array}$$

(19) $$\begin{array}{cc} \Sigma_{T}^{u} & =\left(I-\Sigma_{T}^{p}{\left(\Sigma_{T}^{p}+\Sigma_{T}^{c}\right)}^{-1}\right)\Sigma_{T}^{p}. \end{array}$$

## 6. Experimental Results

**Figure 12:**
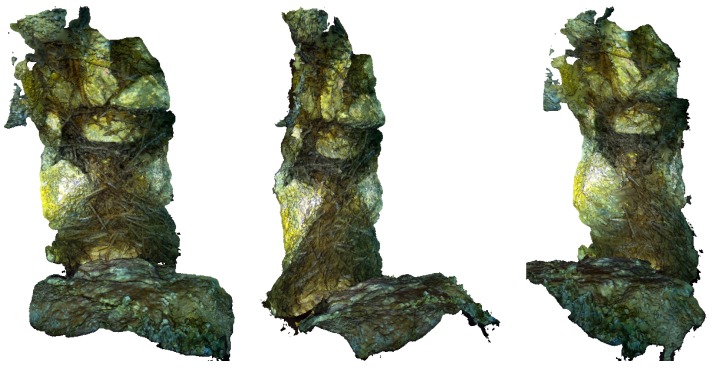
3D reconstructed models using images captured with the handheld system.

The first experiments were carried out to test the hardware platform stability, reliability, and autonomy. Snapshots of the operations for both systems are shown in Figure 4 and Figure 5, whereas examples of the taken images are shown in Figure 6. An underwater site was scanned, and the taken images were processed using photogrammetry techniques to validate the quality of the taken images. We use Agisoft Photoscan [61] to perform the 3D reconstruction. Examples of resulting 3D models are shown in Figure 12. Stereo image synchronization was also validated by observing the relative pose estimation between each pair and comparing it with the stereo calibration extrinsic parameters.

It was desired that the proposed visual odometry method would represent a trade-off between accuracy and runtime, the maximum accuracy being the case for global BA, whereas the fastest runtime was an optimization free visual odometry. Moreover, a performance improvement was expected w.r.t the local optimization method due to a better selection of neighboring observations. Therefore, we analyzed the performance of our method from two points of view: runtime and accuracy.

### 6.1. Runtime Evaluation

We implemented our method using OpenCV [3] bindings in Java. The BA scheme was implemented using the speed optimized BA toolbox proposed in [44]. The image stream processing on the embedded systems including the image quality assessment took around 100 ms per stereo pair to execute. The maximum image acquisition frequency was 3 per second, due to hardware limitations. Therefore, a mid-range laptop computer (with Intel Core i5-7300U CPU @3.50GHz with 16GB RAM) was enough to handle the visual odometry process.

The major improvement that reduced the processing time was the proposed stereo matching method. To demonstrate the time gain, we started by comparing the runtime of our method with an implementation that does not employ any range reduction. The method with range reduction showed an average gain of 72% processing time. Then, we compared this to methods using high-level feature descriptors, in particular, SIFT, SURF, and BRISK. At the same time, we monitored the accuracy for each run. The evaluation was done using the same set of images. In this test, the computational times increased to 342%, 221%, and 142% for SIFT, SURF, and BRISK, respectively. Nevertheless, we noticed a slight gain in accuracy of 1.1% for the average translational error and 0.6% for the average rotational error when using SIFT and SURF, which we do not judge as significant. On the contrary, BRISK performed less accurately, with an increase of 4.1% in average translational error and 0.8% in average rotational error, which is probably due to its sensitivity to water turbidity and dust.

The results above (given in percentages) were more or less consistent across several processing environments, including the running on an RPi computer. Nevertheless, in Table 1, we provide the exact processing times using the same laptop computer mentioned above, applied to full resolution images. The table also shows the percentage of correct matches for each method (our method is not concerned here, as it uses the epipolar geometry to search for matches), which were obtained using the first-order geometric error and a threshold of 0.005. We observe that BRISK features produced less correct matches in underwater images than SIFT and SURF.

**Figure 13:**
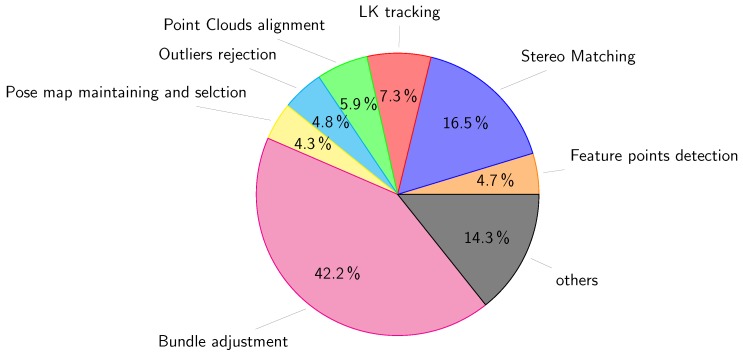
Runtime analysis of the visual odometry system for new pose estimation.

A break-up of the average time required to run the visual odometry is illustrated in Figure 13. It shows that the stereo matching procedure occupies as little as 16.5% of the total runtime, whereas the BA procedure occupies the majority of time with 42.2%. We note that this result is for using five frames in the optimization phase. More frames can be used to improve the accuracy but with a cost of more time complexity. This will be detailed in the next section.

### 6.2. Visual Odometry Evaluation

**Figure 14:**
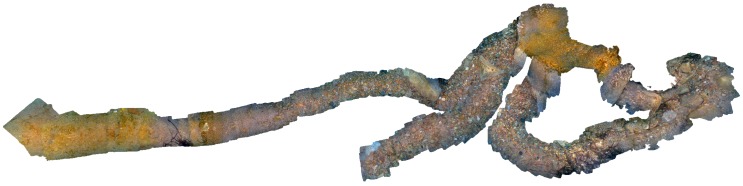
An example of the long trajectory 3D reconstructed model using images captured with the ROV-attached system. Such trajectories were used as ground truths to validate and tune the proposed method.

Unlike terrestrial odometry datasets that come with ground-truth, there is no such option for underwater odometry. Alternatively, to validate the proposed visual odometry method, we conducted an underwater survey using several scenarios, for instance, long trajectories with loop closures or raster scans. We estimated the overtaken trajectories using Agisoft Photoscan which employs a global optimization approach. We used the best available accuracy settings with large numbers of matching points. We considered the estimated trajectories as a reference for comparison in our experiments. An example of a dense reconstruction of such trajectories is shown in Figure 14. It measures around 5 m × 12 m and contains several loop closures. We followed a standard evaluation procedure, as described in [62], where, for all test trajectories, we computed translational and rotational errors for all possible sub-trajectories of one-meter length. The errors were measured as percentages for translation and degrees per meter for rotation.

**Figure 15:**
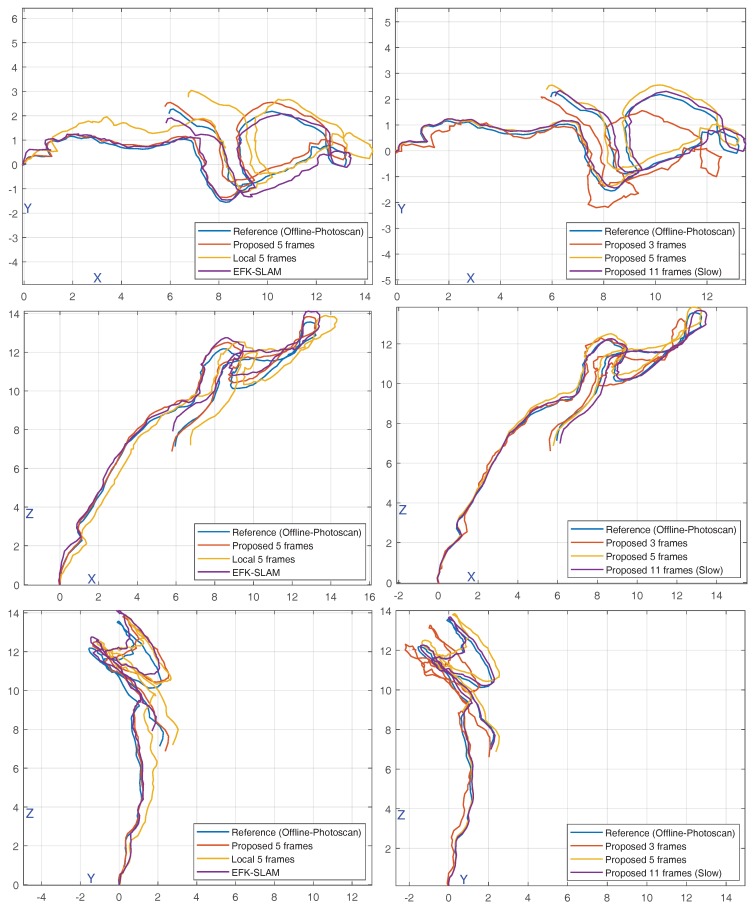
Comparison of trajectory estimation using several methods and parameters. The trajectory obtained using Agisoft Photoscan was considered a reference. It was compared to our method, local optimization using 5 frames, and EFK-SLAM [50] (**left column**). It was also compared to our method in cases where 3, 5, or 11 frames were used for optimization (**right column**). All units are in meters.

We first evaluated the effect of varying the number of frames considered in the optimization phase in our method. Table 2 shows the trajectory errors for using 3, 5, and 11 frames. Although the BA running time for the case of using five frames was around half that of using 11 frames, the accuracy gain was not significant for this latter case. Hence, we found that using five frames is the best accuracy vs. runtime trade-off, and also it is the limit to remain within a real-time performance. The caused drift of 3.8% remained acceptable even for large sites. Figure 15 (right) shows the effect of using different numbers of frames on trajectory estimation for the example shown in Figure 14.

Second, we compared our semi-global BA to three cases—using local BA with the same number of frames, using the underwater EFK-SLAM method [50], and without using any BA. Our method (using five frames) remained ahead of these three variations. From another perspective, although we managed to run a BA-free approach entirely on the third RPi computer in the ROV-attached system. The trajectory drifts were large (with large variance) as shown in Table 2. Trajectory estimation using these methods is shown in Figure 15. From another perspective, although the EFK-SLAM performed better than the local approach, the runtime grew constantly.

## 7. Conclusions and Perspectives

In this work, we introduced several improvements to the current traditional visual odometry approach to serve in the context of underwater surveys. The goal was to adapt the approach to low resource systems. The sparse feature points matching, guided with a rough depth estimation using lightness information, is the main factor associated with most of the gain in computation time compared to sophisticated feature descriptors combined with brute-force matching. Also, using stochastic representation and the selection of frames in the semi-global BA improved the accuracy compared to local BA methods while remaining within real-time limits.

The developed hardware platforms represent efficient low-cost solutions for underwater surveys. The live feedback of image quality and navigation helps to achieve better performance and leads to faster reactions on site. Both systems are flexible for upgrades and modifications; new functionalities can be easily added thanks to the compatible optimized image processing libraries.

Our future perspectives are mainly centered on performing the visual odometry within the system. Further code improvements and parallelism are to be considered. Furthermore, at the time of writing this article, new embedded systems that have double the computational power of the used ones have been released, which makes our objective even closer.

On the other hand, dealing with visual odometry failure is an important challenge, especially in the context of underwater imaging, which is mainly due to bad image quality. The ideas of failing scenarios discussed in this paper can be extended to deal with the problem of interruptions in an obtained trajectory.

## Figures and Tables

**Table 1:** Performance of used feature matching methods regarding processing time and correct matches. The correct matches are defined as having a first-order geometric error [43] (p. 287) less than 0.005.

Method	Detector	Correct Matches (%)	Processing Time (ms)
Ours	Shi-Tomasi [26]	-	220
Stereo matching (no range reduction)	Shi-Tomasi [26]	-	785
SIFT	DoG	49.5	752
SURF	Fast Hessian	48.7	486
BRISK	AGAST [31]	34.3	313

**Table 2:** A comparison of translational and rotational errors for several methods and parameters. The trajectory estimation performed in Agisoft Photoscan was considered to be a reference.

	Translation Error (%)	Rotation Error (deg/m)
Ours (11 frames)—slow	3.8	0.024
Ours (5 frames)	4.3	0.026
Ours (3 frames)	8.2	0.088
EFK-SLAM [50]	5.7	0.032
Local (5 frames)	8.4	0.079
No BA	16.1	0.137

## Abbreviations

The following abbreviations are used in this manuscript:

ROV	Remotely Operated underwater Vehicle
ARM	Advanced RISC Machine
GPS	Global Positioning System
DVL	Doppler Velocity Logs
3D	Three Dimensional
GPU	Graphics Processing Unit
DOF	Degree Of Freedom
BA	Bundle Adjustment
SLAM	Simultaneous Localization And Mapping
RANSAC	RANdom SAmple Consensus
RGB-D	Red Green Blue Depth
RPi	Raspberry Pi
INU	Inertial Navigation Unit
SONAR	SOund Navigation And Ranging
SSS	Side Scan Sonar
SVD	Singular Value Decomposition
CPU	Central Processing Unit
PSD	Positive Semi-Definitive
